# Blood purification therapy prolongs the duration of intensive care unit stay to 14 days or more

**DOI:** 10.1186/2197-425X-3-S1-A761

**Published:** 2015-10-01

**Authors:** H Dohgomori, J Imamura, S Saho, K Kamimura, I Kukita

**Affiliations:** Department of Emergency Medicine, Ryukyu University Hospital, Nishihara-cho, Japan; Department of Emergency Medicine, Kagoshima Medical Center, Kagoshima, Japan

## Introduction

In the intensive care unit (ICU), severely ill or injured patients who undergo specific treatments, including artificial ventilation and blood purification (BT), may require a longer duration of ICU stay than patients not receiving specific treatments.

## Objectives

To clarify the influence of BT on the duration of ICU stay.

## Methods

Patients who received medical treatment in the ICU of Kagoshima Medical Center (Kagoshima, Japan) from August 2014 to December 2014 were enrolled in the present study. APACHE 2 scores were calculated upon admission to ICU. The medical records were retrospectively reviewed for the following: duration of ICU stay, disease severity on admission to ICU shown by APACHE2 score, and necessity for BT. Data obtained from two groups of patients BT+ and BT− were compared and statistically analyzed according to duration of ICU stay. The local ethics committee approved the present study and informed consent was waived.

## Results

A total of 525 patients were admitted to ICU during the study period, and of these, 479 (average age 71.4 years, male/female ratio 303/176) did not undergo BT, while 46 (average age 71.2 years, male/female ratio 35/11) underwent BT. Compared with BT (−) patients, BT (+) patients had significantly higher mean APACHE 2 scores on ICU admission (18.8 vs. 11.1) and longer mean duration of ICU stay (17.1 vs. 4.1 days).

## Conclusions

In Japan, the national health care insurance covers only 14 ICU days for the intensive care by paying additional fee. Therefore, a longer duration of ICU stay incurs additional financial burden on the patient; 135,000 JPN per day for the first 7days and 120000 JPN for the next7 days is paid as the additional fee to hospitals for each ICU patient. However, this is not the case for ICU stay of more than 14 days. In this study, ICU patients undergoing BT were more critical and required intensive care for more than 14 days. Therefore, this may incur additional cost for the patient. To moderate this fee system is thought to be one option for better ICU care.

